# Acknowledgement

**Published:** 2024

We are extremely grateful to the following members of the medical profession from Pakistan and Overseas for sparing their precious time to review the manuscript published in the Issue of March – April 2024.

**Table:** 

Reviewer's Name	(Manuscript Reviewed)	Reviewer's	(Manuscript Reviewed)	Reviewer's Name	(Manuscript Reviewed)
Abrar Ahmed Wagan *Pakistan*		Muhammad Kamran, *Pakistan*		Xie Shi, *China*	
Amjad Khan *South Korea*		Muhammad Naeem Afzal, *Pakistan*		Xin-Wei Liu, *China*	
Basma Al Zahrani *Saudi Arabia*		Musarrat Riaz, *Pakistan*	6	Xin-xin Zhang, *China*	
Chunhong Hou *China*		Nazish Saqlain, *Pakistan*		Yan Gao, *China*	
Ci Liu *China*		Ping Wang, *China*		Yan-qing Deng, *China*	
Daming Yang *China*		Precious Chibuike Chukwuere, *South Africa*		Yaqing Yuan, *China*	
Dashan Chen *China*		Qing-huai Li, *China*		Yong Du, *China*	
Dengsheng Liu *China*		Quan-jin Si, *China*		Yonghui Li, *China*	
Emad Salawati *Saudi Arabia*		Ramesh Kumar, *Pakistan*		Younis Abdelwahab Skaik, *Germany*	2
Faaiz Ali Shah *Pakistan*		Ravi P Upadhyay, *India*		Yu-kun Luo, *China*	
Faisal Nazeer Hussain *Pakistan*	2	Rubina Naqvi, *Pakistan*		Yun Xiao, *China*	
Faraz Shafiq *Pakistan*	2	Saba Sohail, *Pakistan*		Yusra Saleem, *Pakistan*	
Gauthaman Kalamegam *India*		Saeed Ahmed Shaikh, *Pakistan*		Zhihong Li, *China*	
H. Yakup Yakupoglu *Switzerland*		Sahrai Saeed, *Norway*		Zhi-yong Bu, *China*	
Hakan Demirci *Turkey*		Salah A EIMalik, *Saudi Arabia*		Zhonggen Wu, *China*	
Haseeb Mehmood Qadri *Pakistan*	2	Samina Naeem, *Pakistan*		Zhuo Long, *China*	
Huifang Yu *China*		Shulan Yu, *China*			
Hui-ling Xue *China*		Si Jiang, *China*			
Huiyi Ye *China*		Sultan Abdulwadoud Alshoaibi, *Saudi Arabia*			
Huma Mamun Mahmud *UAE*		Syed Irfan Raza, *Pakistan*			
Humaira Zafar *Pakistan*	3	Syed Zulfiqar Ali Shah, *Pakistan*			
Hyun min Lee *China*		Syeda Farah Batool, *Pakistan*			
Ji Jianzhong *China*		Tahir Khaleeq, *United Kingdom*			
Ji Won Seo *China*		Tayyaba Gul Malik, *Pakistan*			
Jinda Yang *China*		Tianjiao Zhu, *China*			
Jing Bi *China*		Urfa Zaryab Mir, *Pakistan*			
Jing-jing Zong *China*		Vincent Adeniyi, *South Africa*			
Lihong Zhang *China*		Weisheng Lu, *China*			
Li-ping Du *China*		Wonkyun Park, *China*			
Liu Hua *China*		Xiao-fang Li, *China*			
Masood Jawaid *Pakistan*	2	Xiao-Jing Zhu, *China*			
Muhammad Ikram Ullah, *Saudi Arabia*		Xiaosong Shen, *China*			

